# Association of Polygenetic Risk Scores Related to Cell Differentiation and Inflammation with Thyroid Cancer Risk and Genetic Interaction with Dietary Intake

**DOI:** 10.3390/cancers13071510

**Published:** 2021-03-25

**Authors:** Sang Shin Song, ShaoKai Huang, Sunmin Park

**Affiliations:** 1 Obesity/Diabetes Research Center, Department of Food and Nutrition, Hoseo University, Asan 31499, Korea; 20195175@vision.hoseo.edu; 2 Department of Bio-Convergence System, Hoseo University, Asan 31499, Korea; 20205623@vision.hoseo.edu

**Keywords:** gene–gene interaction, thyroid cancer, polygenic risk scores, gender, white blood cell, dietary patterns

## Abstract

**Simple Summary:**

Global thyroid cancer incidence is increasing, especially in women. Genetic and environmental factors mutually contribute to its incidence. We aimed to identify genetic variants to influence thyroid cancer risk and determine their interactions with lifestyles in a large city hospital-based cohort (495 thyroid cancer patients and 56,439 control). The best polygenetic model included *DIRC3*_rs6759952, *GAP43*_rs13059137, *NRG1*_rs7834206, *PROM1*_rs72616195, *LRP1B*_rs1369535, and *LOC100507065*_rs11175834, tumorigenesis and cancer cell differentiation-related genes. Their high polygenetic risk scores (PRS) increased thyroid cancer risk by 3.90-fold compared to low-PRS. Thyroid cancer risk was elevated in females, high white blood cell counts, and high energy, low alcohol, and high seaweed intakes by 4.21, 4.03, 7.00, 4.11, and 4.02-fold, respectively. These factors interacted with PRS: the women with high-PRS elevated thyroid cancer risk much among women with high daily energy, seaweeds, and alcohol intake. These results could be applied to personalized nutrition plans to reduce thyroid cancer risk.

**Abstract:**

The incidence of thyroid cancer continues to increase steadily, and this increasing incidence cannot be attributed solely to the overdiagnosis of microcarcinoma or technical advancements in detection methods and may also depend on environmental and genetic factors. However, the impacts and interactions of genetic and environmental factors remain controversial, and they may differ in Eastern and Western countries. The study’s purpose was to identify single nucleotide polymorphisms of genes related to cell differentiation and inflammation to influence thyroid cancer incidence and determine interactions with lifestyles in a large city hospital-based cohort. Genetic variants were selected by genome-wide association study with thyroid cancer participants (case; *n* = 495) and controls without cancers (*n* = 56,439). SNPs having gene–gene interactions were selected by generalized multifactor dimensionality reduction. Polygenic risk scores (PRSs) were generated by summing the number of selected SNP risk alleles. PRSs of the best model included 6 SNPs, that is, *DIRC3*_rs6759952, *GAP43*_rs13059137, *NRG1*_rs7834206, *PROM1*_rs72616195, *LRP1B*_rs1369535, and *LOC100507065*_rs11175834. Participants with a high-PRS had a higher thyroid cancer risk by 3.9-fold than those with a low-PRS. The following variables were related to an increased thyroid cancer risk; female (OR = 4.21), high white blood cell count (OR = 4.03), and high energy (OR = 7.00), low alcohol (OR = 4.11), and high seaweed (OR = 4.02) intakes. These variables also interacted with PRS to influence thyroid cancer risk. Meat/noodle diet patterns interacted with PRSs to increase thyroid cancer risk (*p* = 0.0023). In conclusion, women with a high-PRS associated with cell differentiation and inflammation were at an elevated thyroid cancer risk. Daily energy, seaweeds, and alcohol intake interacted with PRS for thyroid cancer risk. These results could be applied to personalized nutrition plans to reduce the risk of thyroid cancer.

## 1. Introduction

Thyroid cancer is the most common endocrine malignant tumor and has four forms: papillary, follicular, medullary, and anaplastic thyroid carcinoma. The primary subtype is papillary thyroid cancer, which accounts for >85% of all thyroid cancer cases [1]. Thyroid cancer mainly occurs in women aged 30–50. Over the past few years, thyroid cancer has increased more rapidly than any other cancer [2]. Its global incidence rate in women is three times that in men [3], and its incidence is also higher in women in Korea. According to the Global Cancer Observatory’s estimates, the age-standardized incidence of thyroid cancer among women in 2018 was 22.3 per 100,000 persons in the United States, while in 2017, it was 57.2 per 100,000 persons in Korea (https://cancer.go.kr) (accessed on 12 October 2020). The cause of thyroid cancer remains unclear, but it is accepted that genetic and environmental factors affect thyroid cancer risk.

Papillary thyroid cancer is mainly reported to be associated with the missense mutation of *B-raf proto-oncogene* (*BRAF*, serine/threonine kinase) and rearrangement of *RET/PTC1*, *RET/PTC3*, and *neurotrophic tyrosine kinase-1/3* (*NTRK1/3*) genes [4]. RET/PTC fusions are involved in the early etiology of thyroid cancer [4]. A recent genome-wide association study (GWAS) study about thyroid cancer risk has shown PRS with 10 SNPs has thyroid cancer risk by 6.9-fold in 1544 participants having thyroid cancer and 1593 control, and PRS shows stronger prediction power than single genetic variant [5]. However, the genetic variants were selected by statistical significance and not the consideration of gene–gene interaction and etiology [5]. Genetic variants alter gene expression and structure modification to modify gene–gene interaction and gene–environmental interaction to promote or suppress thyroid cancer risk [5]. A recent study has shown the genetic variant–drug interaction in drug treatment in thyroid cancer: Genetic variant rs1512325, located in *nuclear receptor subfamily 3 group C member 2* (*NR3C2*), is associated with remission of thyroid cancer, and the efficacy of venlafaxine treatment is modified by altering serum thyroid-stimulating hormone (TSH) concentrations according to the patients with rs1512325 polymorphism [6]. Genetic variants may also be involved in drug treatment to alter medication efficacy. Thyroid cancer is involved in the differentiation of thyroid tumors, and well-differentiated, poor-differentiated, and undifferentiated tumors in the thyroid gland have different tumor growth and therapeutic outcomes [7].

The major environmental risk factor is exposure to ionizing radiation, as the thyroid gland is more easily irradiated than other tissues because of its anatomic location. Young individuals are more sensitive to radiation and the development of thyroid cancer. Radiation exposure causes somatic mutations by breaking DNA strands, which is considered a cancer risk factor [8], and individuals with a benign thyroid gland history have a significantly higher risk of developing thyroid cancer [9]. A systematic review with nine studies has shown that seven genetic variants in *MGMT*, *XRCC2*, *LIG1*, *ALKBH3*, *ERCC2*, *FOXE1*, *TSHR*, and *NKX2-1* involved in DNA repair pathways interact with ionizing radiation exposure for thyroid cancer risk, and most genetic variants are not associated radiation exposure [7]. Furthermore, the increasing incidence of thyroid cancer over the past decades is consistent with obesity and diabetes increases. However, little is known about the relations between these two conditions and thyroid cancer risk [8]. Estrogen is considered a possible risk factor, given that more than three-quarters of those that contract thyroid cancer are women [9], but the nature of the link between estrogen and thyroid cancer has not been elucidated. These studies suggest thyroid cancer may be associated with increased inflammation and oxidative stress to stimulate DNA damage. The person who is genetically susceptible to inflammation may increase the thyroid cancer risk.

Iodine is an essential trace element and is required for the thyroid hormone (thyroxine,3,5,3′5′-tetraiodothyronine) production and is associated with thyroid disease when intakes are inadequate or excessive [10]. Iodine-induced TSH changes may also increase thyroid cancer risk, and lifestyles influence thyroxine secretion, which is involved either directly or indirectly with thyroid cancer risk [8]. Levels of dietary iodine intake in Korea and Japan are reportedly much higher than in the west due to a higher intake of seaweeds [11]. Furthermore, individual genetic susceptibility can interact with iodine nutritional status and radiation exposure to modulate thyroid cancer incidence [8].

Regarding genetic impacts, disruption in renal carcinoma 3 (*DIRC3*) is involved in thyroid cancer’s pathogenesis in various countries, including Korea [12]. However, other genetic variants have not been consistently reported in different populations. Some individual genetic variants that increase susceptibility to thyroid cancer have been explored in Korea [13]. However, to date, no polygenetic variants have been found to exhibit additive or synergistic effects of genetic variants involved in thyroid cancer etiology on thyroid cancer risk, though the combined effects of single nucleotide polymorphism (SNP) on thyroid cancer risk may be considerable despite their small individual effects. Furthermore, nutritional intakes and dietary patterns may interact with genetic factors, and such interactions between environmental factors and genetic variants have not been studied. We hypothesized that polygenetic variants involved in cell differentiation and inflammation affect thyroid cancer risk and interact with metabolic parameters and nutritional intakes. This hypothesis was evaluated in 56,934 individuals aged > 40 that participated in the urban hospital-based urban cohort of the Korean Genome and Epidemiology Study (KoGES).

## 2. Materials and Methods

### 2.1. Participants

Middle-aged and elderly Korean adults > 40 years old (*n* = 56,934) volunteered to participate in the hospital-based urban cohort of KoGES organized by the Korean Center for Disease and Control during 2004–2013. The Institutional Review Boards approved the KoGES of the Korean National Institute of Health (KBP-2015-055) and Hoseo University (1041231-150811-HR-034-01). Written informed consent was obtained from all participants.

### 2.2. Criteria of Thyroid Cancer and Inclusion/Exclusion Criteria of the Participants

**Figure 1:**
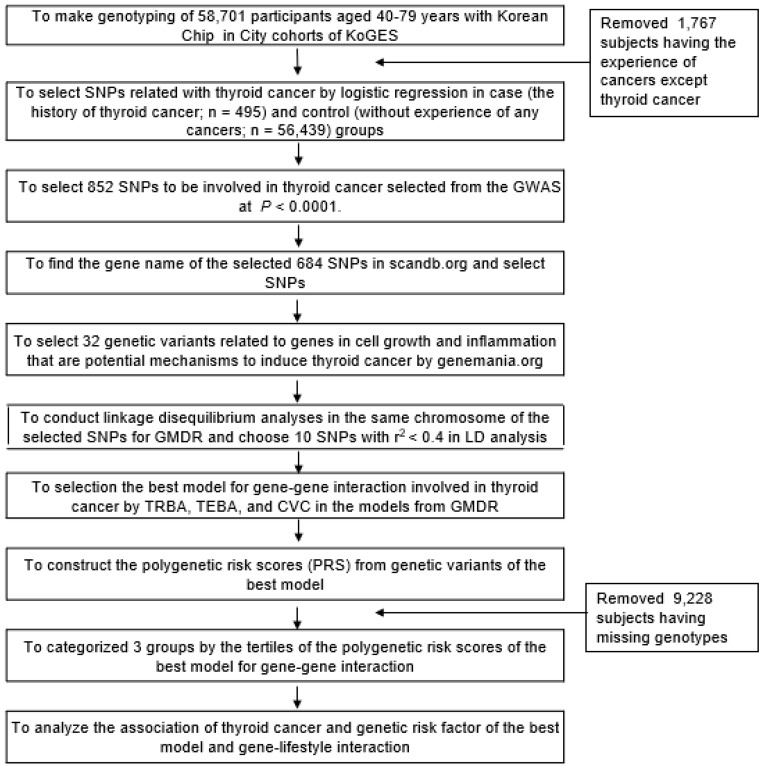
The flow chart for the generation of polygenetic risk scores that influence thyroid cancer risk.

Participants were asked if they had been diagnosed with thyroid cancer by a physician, and those that responded affirmatively were considered to have the disease (case); those that responded negatively were considered the control. Participants were also asked if they had any cancers, and they specified the types of cancers in those who answered “yes”. Among the participants belonged to the Korean hospital-based urban cohort, any history of cancer incidence except thyroid cancer was excluded (*n* = 1767; Figure 1).

### 2.3. Anthropometric and Biochemical Measurements

Information on age, education, income, smoking history, outdoor activities, alcohol consumption, and physical exercise was collected during a health interview [14]. Education level was divided into three groups: less than high school, high school, and ≥college. Household income (USD/month) was categorized into four groups: very low (<$1000), low ($1000–2000), intermediate ($2000–4000), and high (>$4000) [15]. Smoking status was categorized as current smoker, past smoker, and never-smoker [15], and alcohol consumption as nondrinker (0 g daily), mild drinker (0–20 g daily), and moderate drinker (>20 g daily) [15].

A skilled technician measured body weight, height, and waist circumference using a standardized procedure [16]. Body mass index (BMI) was calculated by dividing weight in kilograms by height in meters squared. Blood was collected after a ≥12 h fast (food and drink), and plasma and serum samples were subjected to biochemical analysis [16]. Fasting plasma glucose and serum total cholesterol, HDL, and triglyceride concentrations were measured using a Hitachi 7600 Automatic Analyzer (Hitachi, Tokyo, Japan). White blood cell (WBC) counts were obtained using EDTA-treated blood. Blood pressures were measured on right arms at heart height in a sitting position.

### 2.4. Semiquantitative Food Frequency Questionnaire (SQFFQ) Responses and Dietary Pattern Analysis

Dietary intakes were estimated using an SQFFQ developed and validated for the KoGES [17]. This questionnaire requested information regarding the consumption of food items and details of frequencies and amounts consumed of 106 food items (given assigned serving sizes). The intakes of 23 nutrients were estimated using a Computer-Aided Nutritional Analysis Program 3.0 developed by the Korean Nutrition Society [17].

The 106 food items included were categorized into 29 food groups. These 29 food groups were used as independent variables during the factor analysis to determine dietary patterns using the FACTOR procedure. The number of factors retained in the principle component analysis was determined using eigenvalues of >1.5, and the orthogonal rotation procedure (Varimax) was applied [18]. Dietary factor-loading values of ≥0.40 were considered to indicate significant contributions to dietary patterns. Four distinct dietary factors were selected for the Korean dietary patterns.

### 2.5. Genotyping and Quality Control

Genotype data were provided by the Center for Genome Science at the Korea National Institute of Health. Genomic DNA was extracted from whole blood, and genotypes were determined using a Korean Chip (Affymetrix, Santa Clara, CA, USA). A Korean Chip was developed to study Korean genetic variants and includes known disease-related SNPs [19]. Genotyping accuracy was determined by Bayesian robust linear modeling using the Mahalanobis distance genotyping algorithm [20]. DNA samples were included with the following categories: genotyping accuracies (≥98%), missing genotype call rates (<4%), heterozygosity (<30%), or no gender biases. Genetic variants that met Hardy–Weinberg equilibrium (HWE) inclusion criteria (*p* > 0.05) were included [17].

### 2.6. Identification of the Best Model for Gene–Gene Interactions by Generalized Multifactor Dimensionality Reduction (GMDR) from among the Genetic Variants Selected from the GWAS

The flow chart used to make polygenetic risk scores influencing thyroid cancer risk is shown in Figure 1. Participants were dichotomized into cases (*n* = 495) and controls (*n* = 56,439). GWAS was performed to find genetic variants associated with increased thyroid cancer risk, and genetic variants were selected using the *p* < 0.000001 criteria. The 852 genetic variants were selected, and the corresponding gene names of 684 selected genetic variants were identified using scandb.org (accessed on 7 May 2020). Genes of the SNPs selected for thyroid cancer risk were screened for inflammation and cell growth using genemania.org. The selected 32 SNPs were then checked for linkage disequilibrium (LD) by LD analyses of selected genetic variants in the same chromosomes using Haploview 4.2 in PLINK. SNPs in the same chromosomes were checked for LD. Those with strong LDs were excluded as they provided similar information concerning thyroid cancer risk. The final ten potential genetic variants for the same chromosome’s best model did not show a strong LD correlation (D’ < 0.4). The best model for gene–gene interactions that influenced thyroid cancer risk was selected by trained balanced accuracy (TRBA), test balance accuracy (TEBA), and cross-validation consistency (CVC) using generalized multifactor dimensionality reduction (GMDR) [16]. SNPs in the best model were used to produce polygenetic risk scores. The polygenetic risk score (PRS) for the best gene interaction model was calculated by summing the number of risk alleles of each genetic variant in the selected best model in the model. PRSs were divided into three categories by tertile. A high-PRS indicated a higher number of risk alleles in the best gene interaction model.

### 2.7. Statistical Analyses

The analysis was performed using GPLINK version 2.0 (http://pngu.mgh.harvard.edu/~purcell/plink) (accessed on 16 April 2020) and SAS version 9.3 (SAS Institute, Cary, NC, USA). Using GMDR, the best gene–gene interaction model was selected with a *p*-value of <0.05 by the sign rank test with trained balanced accuracy (TRBA) and testing balanced accuracy (TEBA) with or without adjusting for covariates of age, gender, living area, education, income level, and body mass index [21]. Ten-fold cross-validation was also used to check CVC since the sample size was larger than 1000 [21]. Using the best model determined by GMDR analysis, the risk allele of each SNP in the selected best model was counted as 1 [22]. For example, when the G allele was associated with an increased risk of thyroid cancer, TT, GT, and GG were assigned scores of 0, 1, and 2. PRSs were calculated by summing the risk allele scores of each SNP. The best model with 5 SNPs was divided into three categories (0–3, 4–5, and ≥6) by tertile, that is, into low-, medium-, and high-PRS groups, respectively. The best model containing 6 SNPs was divided into three categories (0–3, 4–6, and ≥7) by tertile into the low-, middle-, and high-PRS groups. Adjusted ORs and 95% CIs for thyroid cancer risk with PRS were calculated after adjusting for covariates. The covariates included were age, gender, residence area, survey year, BMI, education, income, menopause, initial menstruation, smoking, alcohol, energy, physical activity, fat percent intake, and carbohydrate percent intake.

Descriptive statistics for categorical variables (e.g., gender and lifestyle) were calculated based on frequency distributions by PRS tertile (i.e., low-, middle-, and high-PRS). Frequency distributions of categorical variables were analyzed using the chi-squared test. The normality of quantitative variables used in the present study was tested by the Kolmogorov–Smirnov test and QQ plot using Proc Univariate since the sample size was large. The variables had a normal distribution. Means and standard errors were calculated for continuous variables by PRS tertile categories or the presence of thyroid cancer, and their significances of differences were determined by a one-way analysis of variance (ANOVA) with adjustment for covariates. Group multiple comparisons were performed using Tukey’s test. Participants were categorized into high and low intake groups to explain the interaction between PRSs and dietary intake parameters. Two-way ANOVA with main effects and an interaction term was used to investigate interactions between PRSs and lifestyle parameters after adjusting covariates. Statistical significance was accepted for *p* values < 0.05.

## 3. Results

### 3.1. General Characteristics of the Participants According to the Presence of Thyroid Cancer

Table 1 describes the participants’ demographic and clinical characteristics, including 495 cases (having thyroid cancer) and 56,439 controls (having no cancer). The mean age at thyroid cancer diagnosis was 51 years, and age was not associated with thyroid cancer. However, gender had a significant effect, and the risk of thyroid cancer in women was 4.06 times that in men (Table 1). Age at menarche was negatively associated with thyroid cancer incidence (OR = 0.66, *p* < 0.01), but menopause age and pregnancy were not. BMI and waist circumferences had no association with thyroid cancer. However, except for plasma triglyceride concentration, lipid profiles were significantly different between case and control groups (*p* < 0.05). Plasma concentrations of total cholesterol and HDL were significantly lower in the case group than the control, but hypertension and type 2 diabetes were not significantly different in the two groups. Hypothyroidism and hyperthyroidism rates were significantly higher among cases and were positively associated with thyroid cancer by 2.73 and 2.96 times, respectively. White blood cell counts, but not serum CRP concentrations, were also positively associated with thyroid cancer risk by 1.38 times. Education and income levels were not significantly different in the two groups (Table 1).

### 3.2. Nutrient Intakes and Dietary Patterns According to the Presence of Thyroid Cancer

Table 2 describes the nutrient intakes and dietary patterns of cases and controls. Carbohydrate, protein, fat percent intake (*p* = 0.01), exercise (*p* < 0.05), smoking, alcohol, and coffee intake (*p* < 0.001), but not energy and cholesterol intakes, were significantly different between the case and control groups (Table 2). Carbohydrate intake and exercise were positively associated with thyroid cancer by 1.37- and 1.06-fold, respectively, but alcohol and coffee intakes were negatively associated by 0.61- and 0.77-fold, respectively. PCA analysis of dietary patterns showed cases had a prudent diet pattern more than controls and consumed a noodle/meat diet pattern (*p* < 0.001) and Korean balanced diet patterns (*p* = 0.01) less than the control (Table 2). These findings may be associated with reducing smoking and alcohol and coffee consumption, increasing carbohydrate intake and exercise and changing dietary patterns after a thyroid cancer diagnosis. A high noodle/meat diet was negatively associated with the presence of thyroid cancer (*p* < 0.01), but a prudent diet was positively associated with thyroid cancer (*p* < 0.01). It may have been associated with switching from a noodle/meat diet to a prudent diet after receiving a thyroid cancer diagnosis. A traditional balanced diet and a rice-based diet were not associated with thyroid cancer (Table 2).

### 3.3. Association of Genetic Variants For Thyroid Cancer Risk and the Best Model for Gene–Gene Interactions Related to Cell Growth and Inflammation

SNPs associated with thyroid cancer were screened for genes at 10 SNPs were selected after adjusting for age, gender, residence area, survey year, body mass index, daily energy intake, education, and income (Table 3). The selected 10 SNPs were following: rs6759952 of *DIRC3*, rs1369535 of low-density lipoprotein receptor-related protein 1B (*LRP1B*) on chromosome2, rs13059137 of growth-associated protein 43 (*GAP43*) on chromosome3, rs72616195 of prominin1 (*PROM1)* on chromosome4, rs76981250 of pleckstrin and sec7 domain containing 3 (*PSD3)*, rs78371177 of Lysyl oxidase homolog 2 (*LOXL2)*, rs7834206 of neuregulin 1 (*NRG1)* on chromosome8, rs605859 of kirre like nephrin family adhesion molecule 3 (*KIRREL3)* on chromosome11, rs11175834 of *LOC100507065* on chromosome 12, and rs2276010 of mitogen-activated protein kinase 1 (*MAPK1)* on chromosome 22 (Table 3). Each genetic variant was significantly associated within thyroid cancer (ORs = 0.76–1.94; *p* value = 8.51 × 10^−7^ to 0.000899). Genotype frequency distributions met HWE (*p* > 0.05), and their minor allele frequency (MAF) value was > 0.01 (Table 3).

The best model for GMDR for thyroid cancer risk was assessed with genetic variants–genetic variant interaction related to cell growth and inflammation by GMDR. Table 4 shows the ten models generated from the 10 SNPs. Of these models, the best model was selected based on TRBA, TEBA, and CVC values with or without adjusting for the covariates shown in Table 4. The models, including 5 and 6 genetic variants, showed significant associations between gene–gene interactions and thyroid cancer with a sign test *p* value of < 0.05 (Table 4). Models 5 and 6 had the lowest *p*-values among models, and the CVC of both models was 10/10. As a result, the model that included five SNPs, i.e., *DIRC3*_rs6759952, *GAP43*_rs13059137, *NRG1*_rs7834206, *PROM1*_rs72616195, *LRP1B*_rs1369535, and the model that contained six SNPs, i.e., *DIRC3*_rs6759952, *GAP43*_rs13059137, *NRG1*_rs7834206, *PROM1*_rs72616195, *LRP1B*_rs1369535, and *LOC100507065*_rs11175834 were selected as the best models (Table 4). TRBA, TEBA, and CVC values of the 5-SNP model were 0.6498, 0.5494, and 10/10, respectively, after adjustment for age, residence area, and BMI. TRBA, TEBA, and CVC values of the 6-SNP model were 0.6936, 0.5646, and 10/10, respectively, again after adjustment for age, residence area, and BMI.

### 3.4. Association between PRSs Obtained by Summation of Risk Alleles in the Best Model and Thyroid Cancer after Adjustment for Covariates

**Figure 2:**
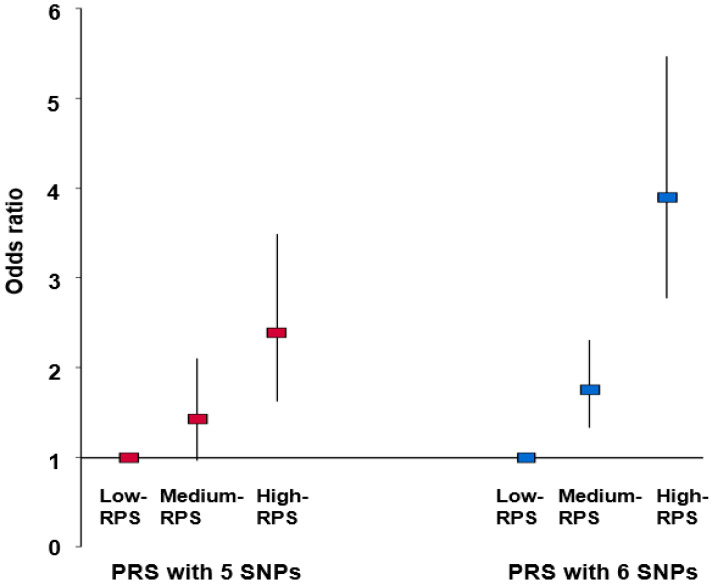
Adjusted odds ratio (OR) and 95% confidence intervals (CIs) of polygenetic risk score (PRS) with 5 SNPs and 6 SNPs generated by SNP–SNP interaction for the thyroid cancer risk. PRS with five SNPs and six SNPs, the best model of GMDR, was calculated by the summation of the number of risk alleles of five and six SNPs, and the calculated PRS were divided into three categories (0–3, 4–5, and ≥6) and (0–3, 4–6, and ≥7) by tertiles, respectively, as the low-PRS, medium-PRS, and high-PRS groups. Adjusted OR was analyzed by logistic regression with the covariates including age, gender, residence area, survey year, body mass index, education, income, menopause, initial menstruation, smoking, alcohol, energy, physical activity, fat percent intake, and carbohydrate percent intake. The reference group was the low-PRS in logistic regression. Red and blue boxes indicated the adjusted ORs for five SNPs and six SNPs, respectively, and the lines through red and blue boxes indicated 95% CI.

The adjusted OR for thyroid cancer in subjects in the high-PRS group as determined using the five SNP model was 2.38 (95% CI: 1.63–3.49) versus the low-PRS group after adjusting covariates (Figure 2). In addition, the adjusted ORs for thyroid cancer in subjects in the high-PRS group as determined using the six SNP model was 3.90 (95% CI: 2.78–5.47). These results indicated that subjects in the high-PRS groups of the five SNPs and six SNPs were at 2.38- and 3.90-fold higher risks of thyroid cancer, respectively, than subjects in the designated low-PRS group. The six SNP model was chosen based on these results rather than the five SNP model for the remaining study.

### 3.5. Interaction between PRSs and General Characteristics and Lifestyles in Thyroid Cancer

**Figure 3:**
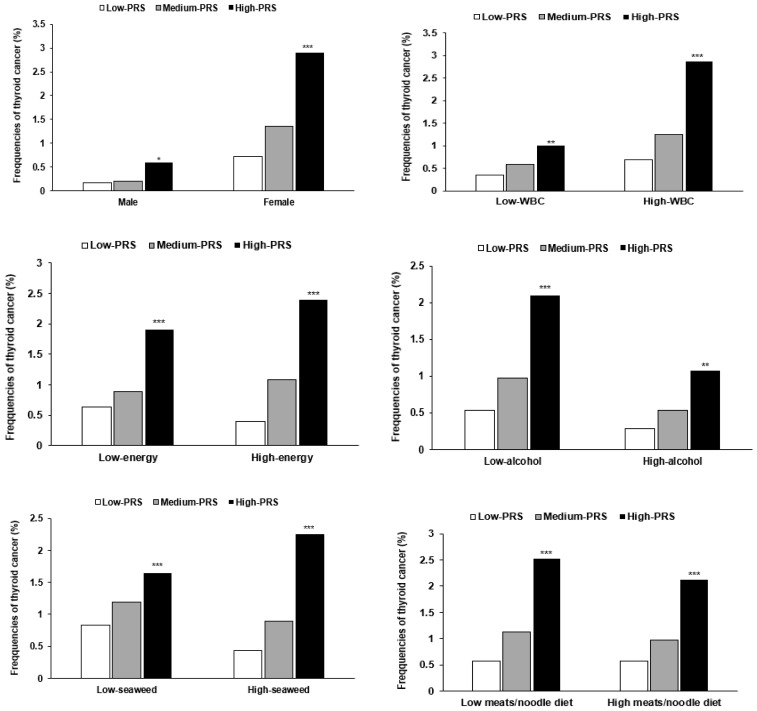
Prevalence of thyroid cancer among subjects in the low-, medium-, and high-PRS groups (determined using the 6 SNP genetic variant–genetic variant interaction model). PRS with 6 SNPs, the best model of GMDR, was divided into 3 categories (0–3, 4–6, and ≥7) by tertiles as the low-PRS, medium-PRS, and high-PRS groups. The nutrient and diet variables were categorized into two groups based on the specified cutoff values. The frequencies of thyroid cancer of PRS groups were calculated in low and high intake groups. A. In subjects categorized by gender. B. In subjects categorized by white blood cell count (WBC, cutoff value: 4 × 10 ^9^/L). C. In subjects categorized by daily energy intake (cutoff value: 100 percent of estimated energy intake). D. In subjects categorized by alcohol intake (cutoff value: 20 g/day). E. In subjects categorized by seaweed intake (cutoff value: 2.65 g/day). F. In subjects with a meats/noodle dietary pattern (cutoff value: 70th percentile). * Significantly different among the PRS groups *p* < 0.05, ** *p* < 0.01, *** at *p* < 0.001.

The interaction between gender and PRS was found to influence thyroid cancer. Women in the high-PRS group of the six SNP model were at 4.21-fold higher risk of thyroid cancer than women in the low-PRS group (*p* < 0.0001, Table 5). For both genders, frequencies of thyroid cancer were significantly higher for subjects with a high-PRS than those with a low-PRS, but this difference was much greater for women (Figure 3A). No significant interaction was found between age and PRS, but the interaction between WBC count and PRS influenced thyroid cancer incidence (*p* = 0.0042; Table 5). Participants with a high white blood cell count had a higher thyroid cancer rate than those with a low white blood cell count regardless of PRS (Figure 3B). Individuals in the high-PRS group with a low white blood cell count had a lower risk of thyroid cancer than those with a high white blood cell count (Table 5, Figure 3B), and individuals in the high-PRS group and a high white blood cell count had a higher risk of thyroid cancer by 4.03-fold than those in the low-PRS group and a high white blood cell count (*p* < 0.001; Table 5).

Energy, alcohol, and seaweed intakes interacted with PRSs to affect thyroid cancer risk (*p* = 0.0143, 0.0014, and 0.0480, respectively). Participants with high energy intake had a higher thyroid cancer rate than those with a low energy intake regardless of PRS (Figure 3C). Those in the high-PRS group with lower energy intakes had a lower risk of thyroid cancer than those with high energy intakes (Table 5, Figure 3C). Subjects in the high-PRS group with high energy intake had a higher risk of thyroid cancer by 7.10 times than those in the low-PRS group with high energy intake (*p* < 0.001; Table 5), and those with mild alcohol intake had a higher thyroid cancer rate than those with moderate alcohol intake regardless of PRS (Figure 3D). Individuals in the high-PRS group with mild alcohol intake had a higher thyroid cancer rate than those with moderate alcohol intake (Table 5, Figure 3D), and those in the high-PRS group with mild alcohol intake had a higher risk of thyroid cancer (by 4.11-fold) than those in the low-PRS group with mild alcohol intake (*p* < 0.001, Table 5). However, the rate of thyroid cancer was higher in the mild alcohol intake group than in the moderate alcohol intake regardless of PRS, which may have been due to reduced alcohol intake after a thyroid cancer diagnosis. Participants with high seaweed intakes had a higher thyroid cancer rate than those with a low seaweed intake regardless of PRS (Figure 3E). Those in the high-PRS group with low seaweed intake had a lower risk of thyroid cancer than those with high seaweed intake (Table 5, Figure 3E), and those in the high-PRS group with high seaweed intake had a higher risk of thyroid cancer by 4.02-fold than those in the low-PRS group with high seaweed intake. (*p* < 0.001) (Table 5). A noodle/meat diet interacted with PRS to influence thyroid cancer risk (*p* = 0.0023). Participants on a low noodle/meat diet had a higher thyroid cancer rate than those on a high noodle/meat diet regardless of PRS (Figure 3F). Subjects in the high-PRS group on a low noodle/meat diet had a higher thyroid cancer rate than those on a high noodle/meat diet (Table 5, Figure 3F). Those in the high-PRS group on a low noodle/meat diet had a higher thyroid cancer rate by 3.97-fold than those in the low-PRS group on a low noodle/meat diet (*p* < 0.001, Table 5).

## 4. Discussion

In the present study, we explored the influences of genetic variants of genes related to cell growth and inflammation on thyroid cancer. GMDR analysis of 32 genetic variants identified by GWAS in the case and control groups identified ten genetic variants related to cell growth and inflammation. We determined the best model for evaluating gene–gene interactions related to cell growth and inflammation that influence thyroid cancer risk from these ten genetic variants. The best SNP–SNP interaction model included 6 SNPs, that is, *DIRC3*_rs6759952, *GAP43*_rs13059137, *NRG1*_rs7834206, *PROM1*_rs72616195, *LRP1B*_ rs1369535, and *LOC100507065*_rs11175834. The PRSs of these six SNPs showed an interaction with gender, white blood cell count, alcohol intake, and nutrient intake, especially energy and seaweed intakes. These results are novel in the context of thyroid cancer risk and could be utilized when advising individuals on personalized nutrition regimes.

*DIRC3* is presumed to have tumor suppressor activity, as it is involved in the production of TSH, a primary factor of thyroid cell growth and function, and indirectly reduces the differentiation of thyroid epithelium, thus promoting thyroid cancer development [12]. Our study also shows that *DIRC3* rs6759952 is positively associated with thyroid cancer, and *DIRC3* rs6759952 polymorphism may reduce TSH to elevate the thyroid hormone, contributing to promoting thyroid tumorigenesis. This finding agrees with Italian, Icelandic, American, Dutch, Spanish, and Polish studies, which reported *DIRC3* rs6759952 showed the strongest association with thyroid cancer in adults [12,23] *GAP43* is a membrane phosphor protein that plays an integrative function in the brain and is expressed at high levels in the developing brain. *GAP43* also promotes thyroid cancer tumorigenesis and tumor progression [24]. However, no study has yet demonstrated an association between *GAP43* SNPs and thyroid cancer. *NRG1* is a glycoprotein that mediates cell-to-cell signaling and is one of the most active members of the epidermal growth factor family [25]. Several studies have reported that *NRG1* is a predisposition to papillary thyroid cancer, but no genetic variant of *NRG1* has been reported about the association with the thyroid cancer risk [13,25]. *PROM1,* also known as *CD133,* is a pentaspan transmembrane glycoprotein that promotes tumor initiation by thyroid cancer stem cells and suppresses cancer cells’ differentiation and helps maintain stem cell properties [26]. *LRP1B* is an endocytic receptor that acts as a tumor suppressor by constraining the invasive behaviors of thyroid cancer and other cancer cells [27]. *LOC100507065* is an RNA gene and is affiliated with the lncRNA class. It is uncharacterized, but its expression is reported to have a relation to sepsis on the skeletal muscle, mir-221 expression effect on the prostate cancer cell line, and RhoGTP dissociation inhibitor 2 effects on UM-UC-3 bladder cancer cells in GEO profiles of NCBI. These reports show that the SNPs’ genes included in the PRS calculations are related to the induction of thyroid cancer by activating epithelial cancer-related cell proliferation and stimulating inflammation. Although individual genetic variants have only modest effects on various diseases, including thyroid cancer, associated risks can increase markedly when combined with polygenetic variants [28]. In the present study, adjusted ORs for thyroid cancer were 3.90 (95% CI: 2.78–5.47) in the high-PRS group as determined by the six SNP model, after adjusting for covariates, as compared with those in the low-PRS group. In an Italian study, the cumulative risk of 11 SNPs, including *DIRC3*, increased thyroid cancer risk [28]. Genetic variants in the *ATM-CHEK2-BRCA1* axis have also been reported to be associated with a predisposition to thyroid cancer [29]. In the present study, PRSs reflected the cumulative risk of thyroid cancer. The present study suggested that *DIRC3*, *GAP43*, and *LRP1B* are involved in tumor suppressor activity to inhibit thyroid tumorigenesis, and their mutation may reduce the suppression of thyroid cancer risk. *PROM1, NRG1,* and *PROM1* are related to stimulating thyroid epithelium to the progression of a thyroid tumor, and their mutation may interact to promote thyroid tumor differentiation and proliferation. Thus, the SNPs of PRS might promote thyroid tumor growth synergistically.

The incidence of thyroid cancer varies between ethnicities, but gender differences are uniformly observed [30]. However, the molecular factors that mediate gender differences have not been well determined. In the present study, women had a significantly higher thyroid cancer rate than men, and the interaction between gender and PRS was found to influence thyroid cancer prevalence. Neutrophil/lymphocyte ratio is positively associated with the platelet–lymphocyte ratio and white blood cell count, and both are prognostic markers of thyroid cancer and thyroiditis [31]. Furthermore, WBC counts and thyroglobulin levels are positively correlated, and elevated thyroglobulin levels indicate poor thyroid cancer survival [32]. These results suggest that high white blood cell counts are positively associated with the thyroid cancer incidence, consistent with our results. Furthermore, we observed that the interaction between PRS and white blood cell counts modulated thyroid cancer risk; for example, individuals in the high-PRS group with a high white blood cell count had a higher risk of thyroid cancer by 4.03-fold than those in the low-PRS group with a high white blood cell count.

High total energy and low polyunsaturated fatty acid intakes have been associated with differentiated thyroid cancer risk [33]. Obesity and protein and carbohydrate consumption higher than those recommended by the World Health Organization have been reported to be risk factors of thyroid cancer [34]. However, in the present study, only carbohydrate intake was significantly associated with thyroid cancer incidence regardless of PRS. Nevertheless, energy intake interacted with PRS to influence thyroid cancer risk. In the high energy intake group, individuals with a high-PRS had a 7.10-fold higher risk of thyroid cancer than those with a low-PRS. Thus, a lower energy intake than the estimated energy requirement (EER) by the Korean Society of Nutrition might be recommended in individuals with high-PRS.

Alcohol consumption is recognized as a risk factor of cancer, but recently a meta-analysis reported an inverse association in thyroid cancer [35,36]. This finding may have arisen because alcohol consumption reduced TSH levels and protected against thyroid cancer development [36]. Unlike the studies in Europeans and Americans, alcohol drinkers are at increased risk of thyroid cancer in Korea [35]. However, in the present study, alcohol intake was negatively associated with thyroid cancer risk and interacted with PRS to reduce cancer risk. Furthermore, subjects with a high-PRS and mild alcohol intake (<20 g/day) had a much higher incidence of thyroid cancer than those with moderate alcohol intake (>20 g/day).

Both low and high iodine intakes can affect thyroid cancer incidence because either can increase serum TSH levels. The role of dietary iodine intake on the prevalence of thyroid cancer remains unclear [8]. French Polynesia, an iodine-deficient region, has one of the highest incidences of thyroid cancer, and those with higher iodine intake in this region are at lower risk [9]. Accordingly, this suggests that relatively low and excessive iodine intakes are associated with thyroid cancer in an iodine-replete region [10]. In Korea and Japan, seaweed is the primary source of dietary iodine, but in Japan, where seaweed consumption is higher than in Korea, no association was found between seaweed consumption and thyroid cancer risk [37]. However, a previous Japanese study reported a four-fold high risk of papillary carcinoma among postmenopausal women who consumed seaweed daily compared to those who ate it ≤two days/week [38]. In China, high seaweed intake was associated with a 1.94-fold increase in thyroid cancer [39]. In the current study, individuals in a high-PRS group with high seaweed intake (>2.65 g/day) had a 4.02-fold higher risk of thyroid cancer than those in the low-PRS group, but those in the low-PRS group had a lower incidence of thyroid cancer than those in the high seaweed intake group. Thus, the association between seaweed intake and thyroid cancer risk is still not clear.

Many studies have shown that dietary patterns can modify thyroid cancer risk, and a western-style dietary pattern may be associated with thyroid cancer [40]. A diet rich in vegetables and fruits has been associated with a lower risk of thyroid cancer [41], whereas starchy foods, sweets, and products rich in salt and fat were associated with thyroid cancer risk. Conversely, milk and dairy products, seafood, and low-fat meat were negatively associated with disease risk [42,43]. However, our findings contradict these findings regarding food intakes and thyroid cancer incidence in the present study. We suggest our results may have been associated with a switch from noodle/meat diets to prudent diets after receiving a thyroid cancer diagnosis. Koreans believe meat consumption compromises cancer treatment and that vegetables and fruits have beneficial effects.

This study’s limitations are as follows: (1) The study’s cross-sectional nature prevented our accessing causal relations. (2) Subjects were recruited from an urban hospital-based cohort, and thus, our results cannot be extended to the Korean population. (3) The diagnosis of thyroid cancer from a physician was self-reported, and the subtypes of thyroid cancer were not defined. However, in Korea, most thyroid cancers are due to papillary thyroid cancer, and the genes included in the 6-SNP PRS-based model were related to papillary thyroid cancer, and thus, our results primarily concern papillary thyroid cancer. (4) Lifestyles were self-reported, and nutrient intakes were based on individual estimates of usual intake. However, nutrient intakes were assessed using a semiquantitative food questionnaire containing 106 commonly consumed food items and was validated using three day-food records over four seasons in KoGES [17]. Nevertheless, this reliance on self-reports may have introduced bias.

## 5. Conclusions

The 6-SNP PRS-based model is composed of *DIRC3*_rs6759952*, GAP43*_rs13059137, *NRG1*_rs7834206, *PROM1*_rs72616195, *LOC100507065*_rs11175834, and *LRP1B*_rs1369535, that is, SNPs related to cell growth and inflammation were found to be positively associated with the risk of thyroid cancer. Furthermore, gender and white blood cell count interacted with PRS, and energy, alcohol, and seaweed intakes and dietary patterns also interacted with PRS to amplify genetic impacts on thyroid cancer risk. Therefore, our findings caution that white blood cell counts should be monitored in subjects with a high-PRS and energy intake (<EER) or seaweed intake and that white blood cell count be monitored for adults of high-PRS to protect against thyroid cancer. Additionally, they show that moderate alcohol intake is not associated with thyroid cancer. These results could be applied to personalized nutrition plans to reduce the risk of thyroid cancer.

## Figures and Tables

**Table 1:** Socioeconomic and metabolic characteristics of the participants according to thyroid cancer.

	Non-Thyroid Cancer (*n* = 56,439)	Thyroid Cancer (*n* = 495)	Adjusted OR (95% CI) ^16^
Age ^1^ (years)	53.6 (53.5, 53.7)	54.1 (53.4, 54.8)	1.140 (0.888–1.463)
Age at diagnosis (years)	-	51.3 ± 0.34	-
Genders (men: N, %)	19,173 (34.9)	50 (10.1) ***	4.056 (2.359–6.974) ***
Initial menstruation age ^2^ (years)	15.1 (15.1, 15.2)	14.9 (14.8, 15.1) *	0.663 (0.510–0.860) **
Menopause age ^3^ (years)	49.3 (49.3, 49.4)	48.8 (48.3, 49.4)	0.971 (0.755–1.247)
Pregnancy experience ^4^ (Yes, %)	35,443 (96.7)	431 (97.1)	1.523 (0.669–3.465)
BMI ^5^ (kg/m^2^)	23.9 (23.9, 23.9)	23.9 (23.6, 24.2)	0.947 (0.734–1.220)
Waist circumference ^6^ (cm)	80.7 (80.7, 80.8)	80.9 (80.5, 81.4)	0.864 (0.598–1.249)
Plasma total cholesterol ^7^ (mg/dL)	197 (197, 198)	189 (186, 193) ***	0.693 (0.543–0.886) **
Plasma HDL ^8^ (mg/dL)	53.8 (53.7, 53.9)	52.3 (51.2, 53.5) *	1.292 (1.061–1.573) *
Plasma triglyceride ^9^ (mg/dL)	125 (124, 126)	125 (117, 132)	1.004 (0.769–1.310)
Hypertension ^10^ (N, %)	13,764 (24.4)	121 (24.4)	1.136 (0.900–1.434)
Type 2 diabetes ^11^ (N, %) ^10^	13,921 (24.7)	117 (23.6)	1.173 (0.935–1.472)
Normal thyroid (N, %)	54,649 (97.2)	433 (89.5)	1
Hypothyroidism Hyperthyroidism	784 (1.39)805 (1.43)	27 (5.58) *** 24 (4.96) ***	2.733 (1.789–4.176) *** 2.962 (1.938–4.525) ***
White blood cell counts ^12^ (10^9^/L)	5.71 (5.69, 5.72)	5.61 (5.46, 5.77)	1.378 (1.122–1.693) **
Plasma hs-CRP ^13^ (ng/mL)	0.139 (0.135, 0.142)	0.147 (0.111, 0.183)	1.440 (0.915–2.268)
Education ^14^ (Number, %) <High schoolHigh school, collegeCollege more	7513 (18.5) 8925 (22.0) 24,125 (59.5)	60 (16.0) 86 (22.9) 229 (61.1)	1.521 (0.970–2.383)
Income ^15^ (Number, %) > 2000 <$2000/year	16,516 (31.0)	137 (29.2)	1.012 (0.706–1.451)
$2000–4000/	23,342 (43.8)	206 (43.8)	
>$4000	13,479 (25.3)	127 (27.0)	

The values represent adjusted means (95% confidence intervals) or number (N; percentage) of the subjects. The cutoff points of the reference were as following: ^1^ <55 years old for age, ^2^ <14 years old for initial menstruation age, ^3^ <50 years old for menopause age, ^4^ no pregnancy experience, ^5^ < 25 kg/m^2^ BMI, ^6^ < 90 cm for men and 85 cm for women waist circumferences, ^7^ <230 mg/dL plasma total cholesterol concentrations, ^8^ >40 mg/dL for men and 50 mg/dL for women plasma HDL cholesterol, ^9^ <150 mg/dL plasma triglyceride concentrations, ^10^ <140 mmHg SBP, 90 mmHg DBP plus hypertension medication, ^11^ <126 mL/dL fasting serum glucose plus diabetic drug intake, ^12^ <4 × 10^9^/L white blood cell counts., ^13^ <0.5 mg/dL serum high sensitive-C-reactive protein (hs-CRP) concentrations, ^14^ high school graduation and ^15^ <$2000/month income. ^16^ Adjusted odds ratio (ORs) and 95% confidence intervals after adjusting for covariates including age, gender, residence area, survey year, BMI, education, income, menopause, initial menstruation, smoking, alcohol, energy, physical activity, fat percent intake, and carbohydrate percent intake. * Significant differences by breast cancer at *p* < 0.05, ** at *p* < 0.01, *** *p* < 0.001.

**Table 2:** Nutrient intake and dietary patterns of the participants according to thyroid cancer presence.

	Non-Thyroid Cancer (*n* = 56,439)	Thyroid Cancer (*n* = 495)	Adjusted OR (95% CI) ^2^
Energy intake ^3^ (%)	96.1 ± 0.14 ^1^	98.1 ± 1.44	0.979 (0.810–1.184)
CHO percent intake ^4^	71.7 ± 0.03	72.6 ± 0.32 **	1.368 (1.020–1.027) *
Protein percent intake ^5^	13.4 ± 0.01	13.1 ± 0.12 **	0.824 (0.649–1.045)
Fat percent intake ^6^	13.9 ± 0.02	13.3 ± 0.25 **	0.781 (0.565–1.079)
Cholesterol intake ^7^	169 ± 0.5	171 ± 5.3	0.900 (0.691–1.173)
Exercise (Number, %) No Yes	25,605 (45.5) 30,676 (54.5)	198 (40.1) * 296 (59.9)	1.358 (1.119–1.646) ** 0.737 (0.607–0.893)
Smoking (Number, %) No Former smoking Smoking	41,096 (73.0) 8893 (15.8) 6303 (11.2)	449 (91.6) *** 32 (6.53) 9 (1.84)	1 0.807 (0.508–1.284)
Alcohol intake (Number, %) No Mild drink (0–20 g)	31,747 (56.3) 1214 (2.15)	378 (76.4) *** 7 (1.41)	1 0.609 (0.471–0.788) **
Moderate drink (≥20 g)	23,478 (41.6)	110 (22.2)	
Coffee intake ^8^ (Number %) Low (<3 g/day)	21,222 (37.6)	233 (47.1) ***	1 0.766 (0.632–0.929) **
Medium (3–16 g/day)	34,650 (61.4)	258 (52.1)	
High (≥16 g/day)	567 (1.0)	4 (0.81)	
Traditional balanced diet ^9^	18,293 (32.4)	132 (26.7) **	0.792 (0.600–1.046)
Prudent diet	18,205 (32.3)	220 (44.4) ***	1.446 (1.139–1.834) **
Noodle/meat diet	18,303 (32,4)	110 (22.2) ***	0.673 (0.506–0.894) **
Rice-based diet	18,263 (32.4)	153 (30.9)	0.840 (0.653–1.082)

^1^ The values represent means ± standard errors or number (percentage) of the subjects. ^2^ Adjusted odds ratio (ORs) after adjusting for covariates including age, gender, residence area, survey year, body mass index, education, income, menopause, initial menstruation, smoking, alcohol, energy, physical activity, fat percent intake, and carbohydrate percent intake in logistic regression models. The cutoff points of the reference were as following: ^3^ < estimated energy intake, ^4^ < 65 energy % carbohydrate (CHO) intake, ^5^ <13 energy % protein intake ^6^ <20 energy % fat intake, and ^7^ <250 mg/day cholesterol intake, ^8^ < 3 g/day coffee drinking, and ^9^ <70th percentile intake of each dietary pattern. * Significant differences by cataract at *p* < 0.05, ** at *p* < 0.01, *** *p* < 0.001.

**Table 3:** The characteristics of the ten genetic variants of genes in thyroid cancer used for the generalized multifactor dimensionality reduction analysis.

Chr ^1^	SNP ^2^	Position	Mi ^3^	Ma ^4^	OR ^5^	^6^*p* Value for ORs	^7^ MAF	^8^*p* Value for HWE	Gene	Functional Consequence	Left Gene	Right Gene
2	rs6759952	218271719	T	C	0.76 (0.64~0.89)	0.000899	0.2491	0.825	*DIRC3*	Intron	*TNP1*	*DIRC3*
2	rs1369535	142636357	G	A	1.27 (1.11~1.45)	0.000488	0.3784	0.062	*LRP1B*	Intron	*MRPS18BP2*	*UBE2V1P14*
3	rs13059137	115347556	C	T	1.29 (1.13~1.48)	0.000261	0.3443	0.261	*GAP43*	Intron	*ZBTB20*	*LSAMP*
4	rs72616195	16043608	T	C	1.29 (1.12~1.47)	0.000263	0.3467	0.407	*PROM1*	Intron	*FGFBP2*	*TAPT1*
8	rs76981250	18761954	T	C	1.77 (1.27~2.45)	0.000635	0.02597	0.744	*PSD3*	Intron	*RPL35P6*	*SH2D4A*
8	rs78371177	23224452	G	A	1.66 (1.25~2.2)	0.000524	0.03784	0.777	*LOXL2*	Intron	*R3HCC1*	*ENTPD4*
8	rs7834206	32406148	G	A	1.46 (1.25~1.69)	8.51 × 10^−7^	0.209	0.549	*NRG1*	utr-5	*NRG1-IT3*	*MST131*
11	rs605859	126407440	C	T	1.51 (1.2~1.89)	0.000403	0.06575	0.081	*KIRREL3*	Intron	*ST3GAL4*	*PRR10*
12	rs11175834	65992636	C	T	1.39 (1.18~1.64)	8.06 × 10^−5^	0.1554	0.238	*LOC100507065*	intron	*MSRB3*	*PCNPP3*
22	rs2276010	22142501	C	T	1.94 (1.38~2.73)	0.000129	0.02019	0.094	*MAPK1*	intron	*YPEL1*	*PPM1F*

^1^ Chromosome; ^2^ Single nucleotide polymorphism; ^3^ Minor alleles; ^4^ Major alleles; ^5^ Odds ratio (lower and upper ends of 95% confidence interval); ^6^ *p*-value for OR after adjusting for age, gender, residence area, survey year, body mass index, daily energy intake, education, and income; ^7^ Minor allele frequency; ^8^ Hardy–Weinberg equilibrium.

**Table 4:** Generalized multifactor dimensionality reduction (GMDR) results of SNP–SNP interaction in the multi-locus of the genes in thyroid cancer.

	No Adjusted				Adjusted for Age, Residence Area, BMI			
Model	TRBA ^1^	TEBA ^2^	*p* Value ^3^	CVC ^5^	TRBA	TEBA	*p* Value ^4^	CVC
*GAP43*_rs13059137	0.5532	0.5157	8 (0.0547)	5/10	0.5538	0.5160	8 (0.0547)	5/10
*NRG1*_rs7834206 *PROM1*_rs72616195	0.5699	0.5155	7 (0.1719)	4/10	0.5708	0.5164	8 (0.0547)	5/10
*NRG1*_rs7834206 *PROM1*_rs72616195 *KIRREL3*_rs605859	0.5847	0.5312	9 (0.0107)	5/10	0.5858	0.5321	9 (0.0107)	5/10
*DIRC3*_rs6759952 *GAP43*_rs13059137 *NRG1*_rs7834206 *PROM1*_rs72616195	0.6121	0.5599	10 (0.0010)	8/10	0.6132	0.5615	10 (0.0010)	8/10
*LRP1B*_rs1369535 plus Model 4	0.6491	0.5516	10 (0.0010)	10/10	0.6498	0.5494	10 (0.0010)	10/10
*LINC02454*_rs11175834 plus model 5	0.6935	0.5625	10 (0.0010)	10/10	0.6936	0.5646	10 (0.0010)	10/10
*KIRREL3*_rs605859 plus model 6	0.7226	0.5275	7 (0.1719)	9/10	0.7223	0.5257	7 (0.1719)	8/10
*LOXL2*_rs78371177 plus model 7	0.7468	0.5212	7 (0.1719)	7/10	0.7468	0.5224	6 (0.3770)	7/10
*PSD3*_rs76981250 plus model 8	0.7701	0.5351	9 (0.0107)	10/10	0.7704	0.5380	9 (0.0107)	10/10
*MAPK1*_rs2276010 plus model 9	0.7849	0.5204	7 (0.1719)	10/10	0.7853	0.5224	7 (0.1719)	10/10

^1^ trained balanced accuracy; ^2^ test balance accuracy; ^3,4^ *p*-value for the significance of the GMDR model by sign test ^3^ without and ^4^ with adjusting for covariates; ^5^ cross-validation consistency; BMI, body mass index.

**Table 5:** Adjusted odds ratios (ORs) for thyroid cancer risk by polygenetic risk scores of the best model (PRS) for genetic variant-lifestyle interaction after covariate adjustments according to lifestyle patterns.

	Low-PRS (*n* = 13,888)	Medium-PRS (*n* = 29,907)	High-PRS (*n* = 3911)	Gene–Nutrient Interaction *p*-Value
Men Women	1	1.277 (0.567–2.872) 1.895 (1.427–2.518)	2.546 (0.880–7.363) 4.212 (2.972–5.969) ***	<0.0001
Young ^1^ Aged	1	1.657 (1.175–2.338) 2.098 (1.368–3.218)	3.182 (2.036–4.973) *** 5.376 (3.254–8.880)	0.2518
Low WBC ^2^ High WBC	1	1.869 (1.078–3.242) 1.810 (1.332–2.459)	3.879 (1.931–7.792) ** 4.032 (2.768–5.871) ***	0.0042
Low energy ^3^ High energy	1	1.376 (0.996–1.899) 3.076 (1.869–5.063)	2.948 (1.942–4.475) ** 7.100 (3.944–12.43) ***	0.0143
Low CHO ^4^ High CHO	1	1.983 (0.866–4.539) 1.803 (1.359–2.393)	5.449 (2.126–13.96) *** 3.816 (2.677–5.441) ***	0.4267
Low protein ^5^ High protein	1	1.703 (1.181–2.456) 1.963 (1.325–2.909)	4.211 (2.699–6.571) *** 3.757 (2.291–6.161) ***	0.1945
Low fat ^6^ High fat	1	1.701 (1.291–2.242) 4.427 (1.342–14.60)	3.777 (2.675–5.333) *** 9.059 (2.433–33.72) ***	0.6550
Low cholesterol ^7^ High cholesterol	1	1.818 (1.357–2.435) 1.838 (0.942–3.586)	3.803 (2.636–5.486) *** 5.107 (2.350–11.10) ***	0.6959
Mild alcohol ^8^ Moderate alcohol	1	1.873 (1.429–2.457) 1.706 (1.011–2.878)	4.105 (2.939–5.734) *** 3.326 (1.688–6.554) ***	0.0014
No exercise Exercise	1	1.674 (1.107–2.530) 1.924 (1.353–2.735)	3.776 (2.255–6.321) *** 4.176 (2.712–6.439) ***	0.4585
Non-smoke Smoker+former	1	1.779 (1.349–2.346) 2.306 (0.786–6.770)	3.877 (2.752–5.461) *** 5.775 (1.616–20.63) ***	0.8004
Low seaweed ^9^ High seaweed	1	1.455 (0.939–2.255) 1.823 (1.395–2.383)	2.052 (0.997–3.838) 4.020 (2.889–5.596) ***	0.0480
Low KBD diet ^10^ High KBD diet	1	1.817 (1.390–2.375) *** 1.775 (1.305–2.413)	3.969 (2.852–5.522) 3.756 (2.555–5.521)	0.4320
Low prudent diet ^10^ High prudent diet	1	1.817 (1.390–2.375) 1.407 (0.998–1.974)	3.969 (2.852–5.522) ** 3.403 (2.236–5.179) ***	0.1113
Low meat/noodle diet ^10^ High meat/noodle diet	1	1.817 (1.390–2.375) 1.989 (1.455–2.718)	3.969 (2.852–5.522) *** 4.408 (3.005–6.466) ***	0.0023
Rice-main diet ^10^ Rice-main diet	1	1.817 (1.390–2.375) 1.953 (1.404–2.717)	3.969 (2.852–5.522) *** 4.370 (2.927–6.524) ***	0.1568

Values represent ORs and 95% confidence intervals. PRS with 6 SNPs, the best model of GMDR, was divided into 3 categories (0–3, 4–6, and ≥7) by tertiles as the low, medium, and high groups. The cutoff points were as following: ^1^ <55 years old, ^2^ <4 × 10^9^/L white blood cell counts, ^3^ <estimated energy intake, ^4^ < 65% carbohydrate (CHO) intake, ^5^ < 13% protein intake, ^6^ < 20% fat intake, ^7^ < 250 mg/d cholesterol intake, and ^8^ 20 g/day alcohol intake, ^9^ 2.65 g/day seaweed intake, ^10^ <70th percentile of each diet pattern, Multiple logistic regression models include the corresponding main effects, interaction terms of SNPs and main effects (energy and nutrient intake), and potential confounders such as age, gender, residence area, survey year, BMI, education, income, menopause, initial menstruation, smoking, alcohol, energy, physical activity, fat percent intake, and carbohydrate percent intake. WBC, white blood cell counts; CHO, carbohydrate; KBD, Korean-style balanced foods. Reference was the low-PRS. * Significantly different from low-PRS in logistic regression analysis at * *p* < 0.05, ** *p* < 0.01, *** *p* < 0.001.

## Data Availability

The data presented in this study are available on request from the corresponding author.

## Abbreviations

SNP: Single nucleotide polymorphism; PRS, Polygenic risk scores; GWAS, genome-wide association studies; Bcl-2, B-cell lymphoma 2; BMI, Body mass index; HWE, Hardy-Weinberg Equilibrium; MAFs, minor allele frequencies; ORs, Odds ratios; CI, Confidence intervals; ANOVA, Analysis of Variance; DIRC3, disruption in renal carcinoma 3 KoGES, Korean Genome and Epidemiology Study; SQFFQ, Semiquantitative food frequency questionnaire; GMDR, generalized multifactor dimensionality reduction; LD, linkage disequilibrium, TRBA, trained balanced accuracy; TEBA, test balance accuracy; CVC, cross-validation consistency; GAP43, growth-associated protein 43; neuregulin 1, NRG1; PROM1, prominin1; LRP1B, low-density lipoprotein receptor-related protein 1B; WBC, White blood cell; EER, estimated energy requirement; SNP, single nucleotide polymorphism; PSD3, Pleckstrin and Sec7 domain containing 3; LOXL2, Lysyl oxidase homolog 2; MAPK1, mitogen-activated protein kinase 1 KIRREL3, kirre like nephrin family adhesion molecule 3.
